# Prevalence and Consequences of Preoperative Weight Loss in Gynecologic Surgery

**DOI:** 10.3390/nu11051094

**Published:** 2019-05-17

**Authors:** Basile Pache, Fabian Grass, Martin Hübner, Amaniel Kefleyesus, Patrice Mathevet, Chahin Achtari

**Affiliations:** 1 Department of Gynecology, Department “Femme-Mère-Enfant”, Lausanne University Hospital, 1011 Lausanne, Switzerland; basile.pache@chuv.ch (B.P.); patrice.mathevet@chuv.ch (P.M.); 2 Department of Visceral Surgery, Lausanne University Hospital, 1011 Lausanne, Switzerland; fabian.grass@chuv.ch (F.G.); martin.hubner@chuv.ch (M.H.); amaniel.kefleyesus@chuv.ch (A.K.)

**Keywords:** enhanced recovery, weight loss, nutrition, surgery, gynecologic/oncology, gynecology, prehabilitation

## Abstract

Preoperative malnutrition and weight loss negatively impact postoperative outcomes in various surgical fields. However, for gynecologic surgery, evidence is still scarce, especially if surgery is performed within enhanced recovery after surgery (ERAS) pathways. This study aimed to assess the prevalence and impact of preoperative weight loss in patients undergoing major gynecologic procedures within a standardized ERAS pathway between October 2013 and January 2017. Out of 339 consecutive patients, 33 (10%) presented significant unintentional preoperative weight loss of more than 5% during the 6 months preceding surgery. These patients were less compliant to the ERAS protocol (>70% of all items: 70% vs. 94%, *p* < 0.001) presented more postoperative overall complications (15/33 (45%) vs. 69/306 (22.5%), *p* = 0.009), and had an increased length of hospital stay (5 ± 4 days vs. 3 ± 2 days, *p* = 0.011). While patients experiencing weight loss underwent more extensive surgical procedures, after multivariate analysis, weight loss ≥5% was retained as an independent risk factor for postoperative complications (OR 2.44; 95% CI 1.00–5.95), and after considering several surrogates for extensive surgery including significant blood loss (OR 2.23; 95% CI 1.15–4.31) as confounders. The results of this study suggest that systematic nutritional screening in ERAS pathways should be implemented.

## 1. Introduction

Preoperative conditioning, recognized as an efficient way to improve surgical outcomes, has been of growing interest in the last decades and endorsed by nutritional societies and perioperative care guidelines [1,2]. Nutritional support strategies have been evaluated and validated for different surgical specialties, including colorectal [3], hepatic [4], or gastric [5] surgery, and for more specific settings, such as surgery for Crohn’s disease [6].

However, in gynecological surgery, evidence is scarce. While specific enhanced recovery after surgery (ERAS) guidelines incorporate multimodal interventions aiming to improve patients’ physical and mental well-being [7], the nutritional aspect focuses on the immediate perioperative period, suggesting limited preoperative fasting, carbohydrate repletion, and early postoperative re-alimentation. However, systematic screening and support strategies for malnourished patients are not specifically recommended. Despite implementation of ERAS guidelines in the gynecological department in 2012, nutritional screening and therapy are not an integral part of daily clinical practice at the present institution. This study thus aimed to assess the prevalence and clinical consequences of preoperative weight loss among patients treated within the standardized ERAS pathway.

## 2. Materials and Methods

### 2.1. Patients

This retrospective study was conducted at Lausanne University Hospital (CHUV), Switzerland, in the gynecologic oncology unit. The study protocol was approved by the local review board (CER-VD # 2017-01996) and was considered as a quality improvement project. Therefore, no written informed consent was requested by the local review board to conduct the study. Results were reported in accordance to the STROBE (Strengthening the Reporting of Observational Studies in Epidemiology) statement.

All consecutive women undergoing elective major (>2 h, general anesthesia, ovarian, uterine, pelvic/lombo-aortic lymphadenectomy, peritonectomy, small-large bowel resection) gynecologic surgery within an ERAS program between October 2013 and January 2017 were included. Patients’ demographics, surgical details, postoperative complications, and length of hospital stay were prospectively recorded by a dedicated clinical nurse. Demographic items (Table 1) included age, American Society of Anesthesiologists (ASA) group, body mass index (BMI), smoking status, diabetes, immunosuppressants usage (i.e., any medication other than chemotherapy causing significant immunosuppression within two weeks prior to surgery (i.e., systemic steroid therapy and specific immunosuppressants, but not non-steroidal anti-inflammatory drugs (NSAIDs) or low dose steroids <10 mg)), length of hospital stay, surgical approach (i.e., minimally invasive versus open surgery), debulking procedure, and duration of procedure. Impact of every individual ERAS item and of overall compliance to ERAS items of >70% were assessed. Surgical complexity scores were modified, according to the original score from Aletti et al. (i.e., ponderation of 12 procedures with individual weighting (max 18 points), allowing gradation for surgical complexity in two groups (low: ≤3 versus high: ≥4)) (Table S1). Cutoffs for continuous variables were set according to institutional standards and ERAS guidelines [8,9].

**Figure 1:**
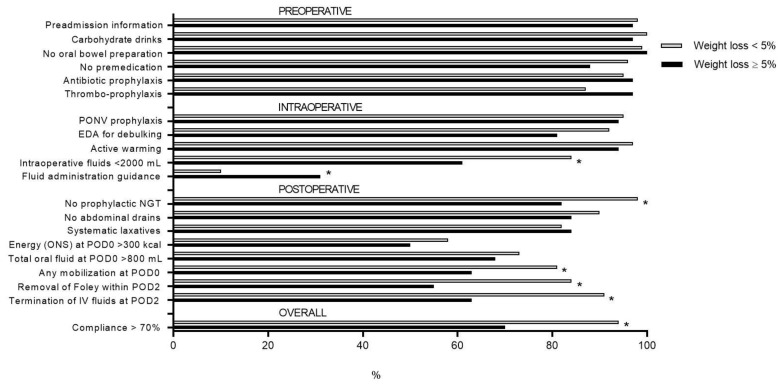
Comparison of compliance to pre-, intra-, and postoperative ERAS items of patients with preoperative weight loss ≥5% within 6 months of surgery (*n* = 33, black bars) and patients with <5% weight loss (*n* = 306, grey bars). ERAS: enhanced recovery after surgery; PONV: postoperative nausea and vomiting prophylaxis; EDA: epidural anesthesia; ONS: postoperative oral nutritional supplements; POD: postoperative day; IV: intravenous. * indicates statistical significance (*p* < 0.05). NGT: Nasogastric tube.

ERAS items for compliance calculation (Figure 1 are divided into three main groups, as previously described [10]; pre-operative: preadmission patient education, preoperative oral carbohydrate treatment, oral bowel preparation, preoperative long-acting sedative medication, antibiotic prophylaxis before incision, thrombosis prophylaxis; intra-operative: postoperative nausea and vomiting (PONV) prophylaxis, epidural if debulking, upper-body forced-air heating cover, total IV volume of fluids intraoperatively with a cut off of 200 mL, fluid administration guidance; and post-operative: nasogastric tube used postoperatively, resection-site drainage, stimulation of gut motility postoperatively, oral nutritional supplements taken on day of surgery with a cut off of 300 kcal, amount of oral fluids taken postoperatively on day of surgery with a cut off of 800 mL, mobilization at all postoperatively on day of surgery, the time to termination of urinary drainage with a cut off of less than 48 h after surgery, and the time to termination of IV fluid infusion with a cut off of less than 48 h after surgery. Compliance with individual ERAS care items and overall ERAS compliance, using a cutoff of 70%, were assessed for each patient [10,11].

A modified surgical complexity score was used to assess the extent of surgery, according to the original score from Aletti et al., (i.e., ponderation of 12 procedures with individual weighting, max. 18 points), allowing gradation for surgical complexity in two groups (low: ≤3 vs. high: ≥4) (Table S1) [12].

### 2.2. Assessment of Weight and Definition of Significant Weight Loss

Weight 6 months prior to surgery was either patient-reported or recorded as per primary care physician. Preoperative body weight was systematically assessed within 2 weeks of surgery during preoperative outpatient visits.

Significant weight loss was defined as unintentional loss of more than 5% during the 6 months prior to surgery [13].

### 2.3. Outcomes

The main outcome was defined as overall postoperative complications, assessed in-hospital and within 30 postoperative days (outpatient control visit) [14]. The secondary outcome was defined as length of hospital stay.

### 2.4. Statistical Analysis

Comparative univariate analysis of patients with ≥5% vs. <5% weight loss was performed with chi-squared test (categorical variables) and *t*-test (continuous variables). Adherence to modifiable ERAS items was calculated in order to assess compliance to individual ERAS items according to previous methodology [10]. In a second step, univariate risk factors (*p* < 0.05) for overall complications were retained to provide adjusted estimations of the odds ratio (OR) through multinominal logistic regression analysis. Data analysis was performed with the Statistical Software for the Social Sciences SPSS Advanced Statistics 22 (IBM Software Group, Chicago, IL, USA).

## 3. Results

### 3.1. Patients

Out of 339 patients, 33 (10%) presented with significant preoperative weight loss of at least 5%., while 14 (42%) of these patients presented a weight loss >10%. On average, these patients lost 7 ± 4 kg of their initial body weight. Demographic and surgical details of both groups (≥5% vs. <5% weight loss) are displayed in Table 1.

While the demographic characteristics of both groups were similar, they differed significantly regarding several surrogates for extensive surgery, including open approach, debulking procedure, duration of operation >180 min, modified Aletti score >3, and intraoperative blood loss >200 mL.

### 3.2. Outcome

**Figure 2:**
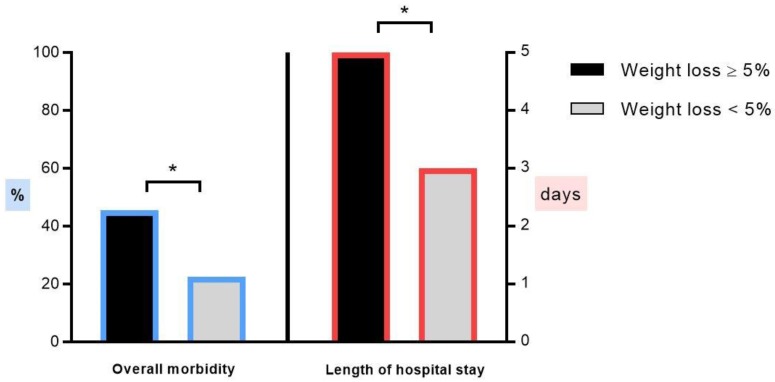
Comparison of postoperative morbidity and length of hospital stay in patients with preoperative weight loss ≥5% within 6 months of surgery (*n* = 33, black bars) and patients with <5% weight loss (*n* = 306, grey bars). Asterisk (*) indicates statistical significance (*p* < 0.05).

Patients with significant weight loss experienced more overall complications (15/33 (45%) vs. 69/306 (22.5%), *p* = 0.009) and a longer length of stay (5 ± 4 days vs. 3 ± 2 days, *p* = 0.011), as illustrated in Figure 2. Overall, surgery-related complications (including intra-operative complications, wound complications, postoperative ileus, and pain) occurred in 41 patients (49%), whereas medical complications occurred in 43 patients (51%), with medical infectious complications in 21 patients (25%).

### 3.3. ERAS Compliance

Compliance to pre-, intra-, and postoperative ERAS items is displayed in Figure 1. Significant differences between the two groups (≥5% vs. <5% weight loss) were found for intraoperative fluid administration <2 L (61% vs. 84%, *p* = 0.003), fluid guidance (31% vs. 10%, *p* = 0.002), avoidance of prophylactic nasogastric tubes (NGTs) postoperatively (82% vs. 98%, *p* < 0.001), mobilization at all on postoperative day (POD) 0 (63% vs. 81%, *p* = 0.029), removal of urinary Foley catheter within 48 h (55% vs. 84%, *p* < 0.001), IV fluid termination within 48 h (62% vs. 91%, *p* < 0.001), and overall ERAS compliance >70% (70% vs. 94%, *p* < 0.001).

### 3.4. Risk Factors for Postoperative Complications

**Figure 3:**
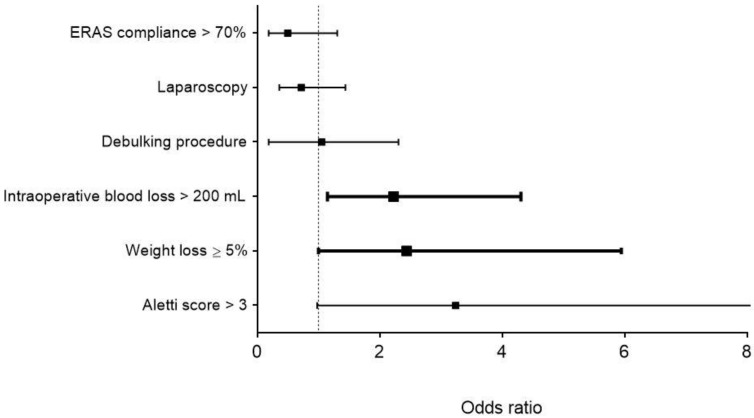
Multivariable analysis of significant univariate demographic and surgical risk factors associated with postoperative complications (*n* = 84). Displayed are odds ratios (black squares) and 95% confidence intervals (black lines). Significant associations (*p* < 0.05) are represented in bold. ERAS: enhanced recovery after surgery.

Multivariable analysis retained significant blood loss >200 mL (OR 2.23; 95% CI 1.15–4.31, *p* = 0.017) and significant weight loss ≥5% (OR 2.44; 95% CI 1.00–5.95, *p* = 0.05) as independent risk factors for postoperative complications (Figure 3).

## 4. Discussion

This study revealed significant preoperative weight loss of >5% in 10% of consecutive unselected patients undergoing a major gynecologic procedure. Furthermore, significant weight loss adversely affected ERAS compliance and length of stay and had a negative impact on the postoperative course, with a 2.5-fold increased risk of adverse events upon multivariable analysis, which considered several surrogates of surgical complexity as confounders.

Malnutrition is a heavy burden for the hospitalized patient, the hospital, and the community and has been comprehensively documented, with a prevalence of up to 73.2% [15]. Similar rates of up to 66% have been described in surgical patients [16], while 20–53% of patients suffering from gynecological cancer present with at least mild malnutrition at diagnosis [17,18]. ERAS protocols in gynecologic surgery aim to improve perioperative nutritional status by focusing on short pre- and postoperative fasting periods and by counterbalancing perioperative insulin resistance [19]. However, the 10% incidence of significant weight loss revealed by the present study calls for the active identification and management of malnourished patients before hospital admission. At a first glance, the observed rate lies within the lower portion of reported ranges. However, no comparative studies focusing on preoperative weight loss within an ERAS setting are available in the gynecologic field. Despite improvements in perioperative care, the present findings support implementation of systematic nutritional screening and, for patients at risk, conditioning through tailored nutritional interventions. This is even more important considering the independent adverse impact of weight loss on postoperative outcomes and duration of hospital stay. Even though not specifically assessed by the present study, further clinical consequences of preoperative weight loss may include an increased risk of surgical site infections [20], lower quality of life (QoL) scores [21], and a significant increase in hospital costs [22,23].

The present study assessed malnutrition solely through significant weight loss. Validated screening tools were not systematically used, which may have led to an underestimation of the actual prevalence of malnutrition in the present cohort. In particular, preoperative food intake was not specifically assessed. Screening with the widespread, and easy to use, nutritional risk score (NRS-2002) could be performed [24]. After identification of patients at risk, more detailed assessment by nutritional specialists may help to define tailored nutritional support strategies [25]. However, a multitude of different screening tools are available and should be used according to institutional standards and local preferences [1].

Type and timing of nutritional support strategies are still a matter of debate [17]. Immunonutrition (combination of amino acids, polyunsaturated fatty acids, and nucleotides or RNA) was recently. No significant difference was found in terms of morbidity at 90 days or survival, but length of stay was shorter in supplemented patients [26]. A prior review evaluating immunonutrition in major abdominal surgery showed reduced mortality, morbidity, and length of hospital stay [27]. A small group of gynecological patients benefited from immunonutrition perioperatively, with a trend toward decreased complications and length of stay (LOS) [24].

Considering recent achievements in perioperative care, the nutritional aspect may be neglected in gynecology, despite encouraging studies [28,29]. In the ERAS guidelines for gynecologic surgery [8,9], there is a real absence of consideration of weight variation. Furthermore, the recently updated guidelines [30], despite recognizing malnutrition as important, do not recommend integrating a prehabilitation program in gynecology. The main reasons for this decision are the heterogeneity of cited studies, varying endpoints, and, most importantly, the lack of large-scale studies in gynecology with consequent extrapolation of results from digestive studies. Just as it has been unattended by the guidelines, the nutritional component of care may have been neglected in our cohort.

There are limitations to this study beyond its retrospective setting. The study cohort is heterogeneous due to the inclusion of consecutive unselected patients. This was accounted for through stratification of surgical complexity. However, both groups differed with regard to extent of surgery, surgical indication, and demographic characteristics. This was addressed by multinominal logistic regression considering all surrogates of surgical extent as confounders and including all univariate risk factors for postoperative complications. Despite standardized care, results of the present single-center study may not be uncritically extrapolated to other settings. Socio-economic factors that may potentially impact the results were not available in the dataset. No formal nutritional screening was performed in the setting of this study, which may have led to an underestimation of malnutrition. Anthropometric measurements to assess, for instance, catabolic state through lean body mass or muscle mass were not performed either. This should be addressed through formal screening by nutritional specialists in future studies.

## 5. Conclusions

In conclusion, preoperative weight loss was identified as an independent risk factor for postoperative complications. Systematic nutritional screening and treatment of malnourished patients before hospital admission should be proposed for gynecologic patients treated within an enhanced recovery pathway.

## Figures and Tables

**Table 1:** Demographics and surgical details.

	All Patients (*n* = 339)	Weight Loss ≥5% (*n* = 33)	Weight Loss <5% (*n* = 306)	*p*
Age (years; mean ± SD)	51 ± 12	53 ± 14	51 ± 12	0.491
>70 years (%)	31 (9)	4 (12)	27 (9)	0.524
ASA group (III–IV; %)	23 (7)	3 (9)	20 (7)	0.481
BMI (kg/m^2^; mean ± SD)	26 ± 6	26 ± 7	26 ± 6	0.798
>25 kg/m^2^	174 (51)	13 (39)	161 (53)	0.149
Weight at surgery (kg; mean ± SD)	70 ± 15	69 ± 17	70 ± 15	0.790
6 months preoperatively Weight loss over 6 months (kg; mean ± SD)	70 ± 15 −0.4 ± 3	76 ± 18 −7 ± 4	70 ± 15 0 ± 2	0.070 **<0.001**
Smoker (%)	64 (19)	4 (12)	60 (20)	0.358
Diabetes (%) Malignancy (%)	16 (5) 132 (39)	0 22 (67)	16 (5) 110 (36)	0.383 **0.001**
Immunosuppression (%)	12 (4)	5 (15)	7 (2)	**0.003**
Minimal invasive approach (%)	244 (72)	17 (52)	227 (74)	**0.008**
Debulking procedure (%)	37 (8)	9 (27)	16 (5)	**<0.001**
Duration of operation (min; mean ± SD)	180 ± 80	240 ± 90	180 ± 80	**0.001**
>180 min	137 (40)	23 (70)	114 (37)	**0.001**
Modified Aletti score (>3; %)	24 (7)	7 (21)	17 (6)	**0.005**
Intraoperative blood loss (mL; mean ± SD)	270 ± 260	320 ± 270	270 ± 260	0.352
>200 mL	192 (57)	20 (61)	172 (56)	0.628

Baseline demographic parameters of patients with (*n* = 33) and without (*n* = 306) preoperative weight loss ≥5% within 6 months before surgery. BMI: Body Mass Index; ASA: American Society of Anesthesiologists; SD: Standard deviation. Age, BMI, duration of operation, and intraoperative blood loss are presented as means ± standard deviation. All others are frequencies and percentages. A bold *p*-value indicates statistical significance (*p* < 0.05).

## Supplementary Materials

The following is available online at https://www.mdpi.com/2072-6643/11/5/1094/s1: Table S1: Surgical complexity scoring system based upon modified Aletti score.
