# Supplementary material for: Prevalence and Consequences of Preoperative Weight Loss in Gynecologic Surgery
Source: Nutrients. 2019 May 17;11(5):1094. doi: 10.3390/nu11051094 (PMC6566827; doi:10.3390/nu11051094)
Supplement: Supplementary file 1 [file nutrients-11-01094-s001.pdf]

Table S1: Surgical complexity scoring system based upon modified Aletti score

| Procedures | Points |
|---|---|
| Total Hysterectomy or uni/bilateral oophorectomy | 1 |
| Omentectomy | 1 |
| Pelvic lymphadenectomy | 1 |
| Para-aortic lymphadenectomy | 1 |
| Small bowel resection | 1 |
| Large bowel resection | 2 |
| Large bowel resection colo-colic anastomosis | 2 |
| Large bowel resection, colo-rectal or colo-anal anastomosis | 3 |
| Splenectomy | 2 |
| Diaphragm stripping/resection | 2 |
| Urological diversion | 2 |
| Liver resection | 2 |
| TOTAL | 20 |

**Table S1**: ponderation of 12 procedures with individual weighting (max 18 points), allowing gradation for surgical complexity in two groups (low: ≤3 versus high: ≥4) derived from Aletti et al.
